# The Prevalence of Drug Habits

**Published:** 1907-10-12

**Authors:** 


### The Prevalence of Drug Habits.

The daily Press has recently again drawn public attention, in connection with an inquest upon a lady who died of an overdose of cocaine, to the deadly results of drug habits. Alcohol and gin habits are terrible, but those of morphia, laudanum, opium, strychnine, arsenic, or cocaine are as bad, if they are not worse. All these habits are carried on in secret, and they are far more common than might be supposed. Those who know this best are the chemists and druggists, for they know how much of each of these drugs is sold for consumption in this way. They cannot help it, for the licensed druggists are not in a position to refuse to supply the drugs, provided the poison-book is properly signed. The general impression of the chemists seems to be that the confirmed drug-drunkards are far more commonly women than men, particularly women of the upper classes with neurotic temperaments. Morphinomania is probably more common than the others; but the cocaine habit is almost as widespread. The commonest methods of taking cocaine are by inhalation as snuff, and by rubbing a liquid preparation into the gums. There is no need here to discuss the symptoms produced, nor the shattering of all the systems that results. The amount of the drug that is tolerated increases with habituation, but the risk of an overdose is always present. The habit once formed can scarcely ever be cured; or, if one drug habit is cured, the patient almost immediately adopts another. The effects of the habits, not only upon the patients themselves, but also upon their husbands, wives, families, relations, and friends, are so terrible that, quite apart from the risk of death from an overdose, there can be no two opinions about the desirability of preventing the disease. It is a habit that, if it cannot be cured, ought to be made one more difficult to acquire.

The following is a remedy suggested by a chemist, quoted from the *Tribune*:—

"The only remedy is to make the sale of poisons illegal without producing a doctor's prescription of recent date. As the law now stands, a chemist may serve small quantities of drugs to anyone, provided he makes them sign his book. The dating of a prescription is an important point, and it should be made illegal to serve poisonous drugs on a prescription more than, say, a month or two months old. The commonest expedient of the drug-taker for obtaining supplies is by means of a prescription which, being undated, as they almost invariably are, can be and are used for years and handed from one to another."

We are not responsible for the English in the above quotation, but we feel sure that the remedy proposed is a sound one. If a time-limit were set to prescriptions for poisons—and we may here add that there should be a time-limit for all prescriptions— the result would be a benefit to the profession and a safeguard to the public. We hope that the matter will be taken up by those in authority. When the suggestion that prescriptions should have time-limits comes from the medical profession itself, the public is naturally apt to think that it arises from interested motives; but in connection with setting difficulties in the way of these terrible drug habits no such motive can be adduced, more especially as the suggestion now comes from one whose self-interest would best be served by leaving things as they are. There is urgent need for action in the matter, and there appears to be no great difficulties in the way of amending or altering the law in such a manner that the facilities now placed in the way of the drug-drunkard may be abolished.

